# Reduction in Remnant Cholesterol in Obese Individuals After Bariatric Surgery with Gastric Bypass or Sleeve Gastrectomy

**DOI:** 10.3390/nu17010189

**Published:** 2025-01-05

**Authors:** Jan O. Aaseth, Kjetil Retterstøl, Helge Rootwelt, Per G. Farup

**Affiliations:** 1 Department of Research, Innlandet Hospital Trust, P.O. Box 104, N-2381 Brumunddal, Norway; per.farup@ntnu.no; 2 Department of Health and Nursing Science, Faculty of Health and Social Sciences, Inland Norway University of Applied Sciences, N-2418 Elverum, Norway; 3 Department of Nutrition, Institute of Basic Medical Sciences, University of Oslo, N-0317 Oslo, Norway; kjetil.retterstol@medisin.uio.no; 4 Lipid Clinic, Oslo University Hospital, N-0424 Oslo, Norway; 5 Department of Medical Biochemistry, Oslo University Hospital, N-0424 Oslo, Norway; hrootwel@ous-hf.no; 6 Department of Clinical and Molecular Medicine, Faculty of Medicine and Health Sciences, Norwegian University of Science and Technology, N-7491 Trondheim, Norway

**Keywords:** dyslipidemia, cholesterol, remnant cholesterol, LDL, HbA1c, morbid obesity, gastric bypass, gastric sleeve

## Abstract

**Background:** The effectiveness of bariatric surgery in reducing remnant cholesterol (RC) levels, particularly when obesity is accompanied by elevated glycated hemoglobin (HbA1c), is insufficiently investigated. In this study, we aimed to examine the impacts of two common bariatric procedures, Roux-en-Y gastric bypass (RYGB) and sleeve gastrectomy (SG), as regards their effects on RC and HbA1c levels. **Methods:** Adult morbidly obese subjects were included and assigned to receive either RYGB or SG. The levels of RC and HbA1c were determined 6 and 12 months after surgery and compared to preoperative levels to assess the efficacy of these surgical methods. In the statistical evaluation of covariations between RC and other biomarkers, previously determined C-reactive protein (CRP), triglycerides, apolipoprotein B, apolipoprotein A1, and low- and high-density lipoprotein cholesterol 6 and 12 months after surgery were included. A linear mixed regression model for repeated analyses was used. **Results:** The RC levels were markedly reduced both after RYGB and SG but without significant differences between the RYGB and the SG surgery. Furthermore, the RC values were strongly associated with the levels of CRP and HbA1c. **Conclusions:** A significant lowering of RC values after bariatric surgery appeared paralleled by concomitant reductions in HbA1c values and CRP levels. Together, these effects lead to a lower risk of cardiovascular disease.

## 1. Introduction

Obesity has increased in prevalence throughout the world over the past decades and is often accompanied by dyslipidemia and diabetes mellitus type 2 (T2DM) [1]. Several studies have revealed an increased risk of cardiovascular disease (CVD) in obese patients [2,3]. Bariatric surgery, which is considered an efficient treatment, is reserved for patients with severe obesity, defined as a body mass index (BMI) > 40 kg/m^2^ or BMI > 35 kg/m^2^ in the presence of severe comorbidities such as T2DM [4]. In Norway and other countries in the Western world, the two most common methods for bariatric surgery are Roux-en-Y gastric bypass (RYGB) and sleeve gastrectomy (SG). RYGB is a procedure that involves the creation of a small stomach pouch and the connection of this pouch to the small intestine to obtain a bypass of the major part of the stomach and duodenum. Sleeve gastrectomy involves surgical removal of about 80% of the stomach, leaving a tube-shaped, very small stomach postoperatively. Standardized laparoscopic surgery is routinely used for both RYGB and SG. Both these methods for bariatric surgery result in substantial weight loss and reduced mortality from CVD [5].

A critical risk factor for CVD is atherosclerosis driven by dysregulated lipid metabolism with high levels of low-density cholesterol (LDL-c). However, despite the achievement of recommended levels of LDL-c, a residual risk of CVD represents a major problem. Remnant cholesterol (RC) has, in recent years, emerged as an additional risk factor for CVD [6]. RC is a component of total cholesterol (TC), which is composed of lipoprotein particles rich in triglycerides (TG) that, in the fasting state, consist of very low-density lipoprotein (VLDL) and intermediate-density lipoprotein (IDL). RC is usually quantified by use of the formula RC = TC − LDL-c − HDL-c. Although its concentration can be determined by direct methods such as ultracentrifugation, such measurements are costly and not easily available. Thus, the calculation method is preferred both clinically and in research [6].

The relationship between RC, atherosclerosis, and CVD appears well established. Castañer et al. revealed that RC is associated with CVD outcomes [7]. A study on participants diagnosed with diabetes from the NHANES 1999–2018 indicated a U-shaped association between RC and cardiovascular deaths [8]. However, a later study of a general population in Copenhagen suggested a causal relationship between RC elevation, low-intensity inflammation, and an increased risk of ischemic heart disease (IHD) [9]. Furthermore, a prospective study of the Danish general population showed that reducing RC levels by about 0.8 mmol/L could decrease recurrent major CVD events by 20% [10]. Varbo et al. [11] reported that the elevated risk of CVD in obesity could partly be ascribed to elevated levels of RC. These studies highlight the critical role of RC in the development of atherosclerotic CVD, not least in obese individuals. Both lifestyle interventions and drug therapies have proven efficacy in reducing RC levels. Unfortunately, these strategies appear insufficient in advanced cases [12].

To the knowledge of the authors, the impact of bariatric surgery on RC has not yet been investigated, except for a six-month follow-up after gastric bypass conducted by Zhao et al. [13]. In the present study, which represents a follow-up of our previous study on circulating lipids after bariatric surgery [14], we aimed to examine the impacts of the two most common bariatric procedures, RYGB and SG, on RC. Furthermore, since the associations between RC and the syndrome triad of body weight, inflammation, and diabetes are still insufficiently explored, the present investigation aimed at providing improved insight into possible associations between RC and these characteristics.

## 2. Materials and Methods

The present retrospective cohort study is a secondary analysis of data collected in the cohort study MO-BiPS (Morbid Obesity—BioPsychoSocial impacts) [15]. Inclusion criteria for surgery were age 18–60 years and morbid obesity, defined as BMI > 40 kg/m^2^ or BMI > 35 kg/m^2^ with obesity-related comorbidity at the time of referral. Exclusion criteria were severe somatic and psychiatric disorders not related to obesity, in addition to alcohol or drug addiction and previous major abdominal surgery.

Analyses of blood plasma samples collected from the cubital vein of the participants in the cohort study MO-BiPS [15] have been used in the present retrospective study. The participants in the MO-BiPS study went through a preoperative behavioral intervention for half a year with guidance on a diet and adapted physical training [15]. Thus, they attended an eight-week course with weekly visits preoperatively. During the last 21 days before bariatric surgery, they were given a specified low-calorie diet with an energy intake of 3765 kJ/day [15]. The surgery was performed either as RYGB or SG, the allocation to the operative method being conducted by the surgeon in agreement with the participant. Postoperatively, the nutrition was mostly based on liquids in the first week, followed by progress to mashed food during the subsequent weeks. From one month after surgery, the nutritional recommendations for the participants were principally the same as those given to the general population, with a relatively low intake of simple carbohydrates and fat. In addition, the participants were given recommendations for lifelong supplementation with vitamins and minerals [15].

As previously described [14], there were clinical follow-ups with clinical examination and blood samplings immediately before and six and 12 months after surgery, the first blood sampling being conducted after 12 h of fasting shortly before surgery. As for the subsequent blood sampling six and 12 months postoperatively, information on fasting had been given. Blood samples were drawn from the cubital vein and centrifuged at 2200× *g* for 10 min at 4 °C. In the present study, data from these visits were used. Concentrations of HbA_1c_ were determined shortly after blood sampling by use of Cobas c501 (Roche Diagnostics, Chicago, USA). Previously determined levels of CRP, apoB, apoA1, LDL-c, and HDL-c were used in the statistical analyses. RC levels were calculated as the total cholesterol amount minus the LDL-c and HDL-c. The plasma analyses of cholesterol, LDL-c, and HDL-c were conducted at the local laboratory of Innlandet Hospital by use of the Cobas c501 analyzer, whereas the levels of apoB and apoA1 were determined at the Department of Medical Biochemistry, Oslo University Hospital, on the Cobas800 c702 instrument [14].

*Statistics.* The normality of the residuals was assessed by inspection of the QQ plots (quantile–quantile plots). The plots showed a satisfactory approximation to normality. Data are presented by mean and standard deviation. The laboratory results obtained 6 and 12 months after surgery were combined in the analytical comparison with the results before surgery. A linear mixed regression model was used for the multivariable analyses. The results were reported as estimated marginal means or estimated coefficients (B-values) with 95% confidence intervals and *p*-values. Because of multiple testing, *p*-values < 0.01 were judged as statistically significant. Statistics were performed with IBM SPSS Statistics for Windows, version 29.0 (IBM Corp., Armonk, NY, USA).

*Ethics.* The participants gave written informed consent before their inclusion. This study was approved by The Norwegian Regional Ethical Committee for Medical and Health Research, P.O. Box 1130, Blindern, 0318 Oslo, Norway (reference number 2012/1394 with the amendment of 12 April 2023), and it was carried out in accordance with the Declaration of Helsinki.

## 3. Results

A total of 121 participants underwent bariatric surgery, of whom 111 had blood samples before and after surgery and were included in the present study. Baseline characteristics were: men/women: 88 (79%)/23 (21%); BMI 38.8 (SD 3.8) kg/m^2^; type of surgery: RYGB/SG 90 (81%)/21 (19%); diabetes (T2DM), 19 (17%); HbA1c 5.48 (SD 0.87) (pct); CRP 4.41 (SD 3.92) mg/L; TG 1.33 (SD 0.52) nmol/L; TC 4.36 (SD 0.88) mmol/L; HDL-c 1.11 (SD 0.30) mmol/L; and LDL-c 2.78 (SD 0.82) mmol/L. Our previous paper [14] details participant characteristics, changes in the lipids in the two types of surgery, and associations between the lipids and BMI, diabetes, age, and sex. The average postoperative weight loss during the first year was 32 kg from a preoperative average body weight of 123 kg.

This study disclosed a significant reduction in the RC values and the HbA1c values after bariatric surgery in all participants and in both surgical groups (Table 1) without significant differences between the groups.

Associations between the lipids and BMI, CRP, and HbA1c are given in Table 2. TG and RC were significantly associated with CRP and HbA1c. No such associations were found for ApoA1, ApoB, or LDL-c. However, the LDL-c/HDL-c-ratio was associated with CRP and BMI.

Furthermore, as seen in Table 3, RC was highly significantly associated with all the lipid markers analyzed except for ApoA1.

## 4. Discussion

In this study in which severely obese participants underwent bariatric surgery with RYGB or SG, we found that the average weight loss during the first year after surgery, which amounted to 32 kg (26%) [14], was accompanied by a significant reduction in circulating RC values. Furthermore, we disclosed an association between the reduced RC values and reduced inflammation as assessed by CRP. The declining RC values were also associated with the reduction in HbA1c, this reduction being in accordance with the previously reported reduction in T2DM and CVD risk postoperatively [16,17]. Inflammation, as well as raised values for HbA1c and LDL, are well-known predictors of CVD [17,18].

Surprisingly, the reduced RC values during the observation period seemed not to be directly related to the surgery-induced reduced BMI or weight loss (see Table 2); the changed RC values appeared associated with metabolic variables (HbA1c and CRP) and thus presumably dependent on a changed lifestyle postoperatively. Postoperative dietary changes and/or hormonal changes may have contributed to improved glycemic control (HbA1c). Dietary changes with caloric restriction may have played a role in both the RC and the HbA1c reduction. Furthermore, bariatric surgery may, by accelerating the transit of food to the gut, increase the release of incretins and other gut hormones and may also influence HbA1c and CRP values through its ability to alter the gut microbiota [17].

Although we could observe an association between RC and LDL-c, no significant associations could be traced for the LDL-c values toward HbA1c or CRP values. Based on these observations, it is tempting to propose RC as a more relevant risk factor than LDL-c for CVD [18], at least in overweight individuals, where a significant proportion is known to suffer from insulin resistance and low-grade inflammation. Previous studies have also reported increased RC to represent a significant risk factor for CVD [19,20,21,22,23]. The mechanisms and implications of the postoperative RC reduction are not fully understood and call for further research. Improvements in liver functions postoperatively might have contributed to the reduction in RC. Regardless of the mechanisms involved, the changes observed in RC might have important implications as regards a reduced risk of CVD and related societal costs.

It is known that elevated TG, as well as increased RC levels, are often caused by obesity and suboptimal lifestyle habits, such as diets high in refined carbohydrates and saturated fats combined with a sedentary lifestyle [23]. The initial step recommended for managing elevated RC is to adjust the diet and achieve weight loss, i.e., by increasing intake of fiber-rich foods such as fruits, vegetables, and whole grains. Obesity-reducing drugs are advised in selected cases [24]. It is known that classic lipid-lowering drugs, such as statins, can all lower RC levels to some extent [7]. Promising results have been obtained by adding the drug ezetimibe to statin therapy in severe cases [25]. Bariatric surgery is only recommended in cases of severe, so-called morbid obesity.

Insulin resistance with elevation of HbA1c, often precipitating T2DM, can have a significant impact on lipid metabolism and RC values. Insulin resistance is one of the mechanisms behind high TG levels in obese individuals with cardiometabolic syndrome or T2DM and can increase VLDL production by inhibiting apolipoprotein B degradation, leading also to elevated RC levels [23]. Therefore, managing insulin resistance through lifestyle changes and appropriate medication may help reduce RC levels and prevent or delay the development of CVD.

### Limitations

A limitation of the present study is the low number of study participants (n = 111) and the predominance of women (79%) in the material. The observations on a significant association between remnant cholesterol with HbA1c and also with CRP in these obese individuals undergoing bariatric surgery are not necessarily generalizable to other populations. The limited sample size makes extrapolation of the results difficult. The age range in the material is also limited. In spite of this limitation, the observations provide new and hypothesis-generating information that deserves follow-up studies in more comprehensive materials.

## 5. Conclusions

In this study, we have shown that the weight loss following bariatric surgery with RYGB or SG, which protects against cardiovascular mortality, is accompanied by reductions in the cardiometabolic risk factors RC, HbA1c, and CRP. Bariatric surgery is reserved for selected cases with severe obesity, while lifestyle modifications combined with lipid-lowering treatments can act by reducing circulating levels of RC. Future studies on efficient treatments for RC lowering and more detailed information on the relationships between RC values and CVD are needed.

## Figures and Tables

**Table 1:** Remnant cholesterol (RC, mmol/L) and HbA1c (%) pre- and postoperatively in all participants and divided into types of surgery, either Roux-en-Y gastric bypass (RYGB) or sleeve gastrectomy (SG), with comparisons between the groups. The analyses were performed with a linear mixed regression model adjusted for sex, age, type of surgery, and point of time. The changes are reported as estimated marginal means with 95% confidence intervals and *p*-values. Statistically significant *p*-values are given in boldfaced type.

Dependent Variables	Before Surgery	After Surgery	Changes	95% CI	*p*-Value
**RC**					
All	0.466	0.204	−0.262	−0.293; −0.230	**<0.001**
RYGB	0.499	0.228	−0.272	−0.306; −0.237	**<0.001**
SG	0.415	0.195	−0.220	−0.292; −0.147	**<0.001**
**HbA1c**					
All	5.501	5.172	−0.330	−0.402; −0.258	**<0.001**
RYGB	5.460	5.115	−0.344	−0.424; −0.265	**<0.001**
SG	5.517	5.253	−0.264	−0.432; −0.097	**0.002**

RC: change difference: RYGB—Sleeve: −0.052; 95%CI: −0.132; 0.029; *p* = 0.207. HbA1c: change difference: RYGB—Sleeve: −0.080; 95%CI: −0.265; 0.105; *p* = 0.852.

**Table 2:** Associations between the lipids/lipoproteins, one at a time, and BMI, CRP, HbA1c, sex, age, type of surgery, and point of time, and all simultaneously. The analyses were performed with a linear mixed regression model and reported as estimated coefficients (B-values) with 95% confidence intervals and *p*-values. Because of multiple testing, *p*-values < 0.01 were judged as statistically significant. Statistically significant *p*-values are given in boldfaced type.

Lipids	BMI B-Value (95% CI) *p*-Value	CRP B-Value (95% CI) *p*-Value	HbA1c B-Value (95% CI) *p*-Value
Triglycerides (mmol/L)	0.013 (−0.003; 0.029) *p* = 0.100	0.023 (0.007; 0.039) ***p* = 0.005**	0.180 (0.104; 0.256) ***p* < 0.001**
Cholesterol (mmol/L)	0.018 (−0.012; 0.048) *p* = 0.235	0.023 (−0.009; 0.055) *p* = 0.159	0.133 (−0.010; 0.277) *p* = 0.068
HDL-c (mmol/L)	−0.013 (−0.023; −0.003) *p* = 0.013	−0.001 (−0.010; 0.009) *p* = 0.872	−0.015 (−0.068; 0.038) *p* = 0.584
Non-HDL-c (mmol/L)	0.030 (0.000; 0.059) *p* = 0.049	0.030 (−0.001; 0.060) *p* = 0.060	0.143 (0.000; 0.287) *p* = 0.050
LDL-c (mmol/L)	0.026 (−0.002; 0.054) *p* = 0.070	0.017 (−0.012; 0.047) *p* = 0.243	0.100 (−0.035; 0.234;) *p* = 0.145
LDL-c/HDL-c ratio	0.049 (0.017; 0.081) ***p* = 0.003**	0.060 (0.029; 0.091) ***p* < 0.001**	0.127 (−0.033; 0.287) *p* = 0.119
ApoB	0.006 (−0.001; 0.014) *p* = 0.103	0.006 (−0.001; 0.014) *p* = 0.101	0.035 (−0.001; 0.072) *p* = 0.058
ApoA1	−0.003 (−0.011; 0.004) *p* = 0.388	0.003 (−0.005; 0.010) *p* = 0.516	0.015 (−0.025; 0.054) *p* = 0.471
ApoB/ApoA1 ratio	0.009 (0.001; 0.016) *p* = 0.020	0.009 (0.002; 0.017) *p* = 0.011	0.017 (−0.019; 0.053) *p* = 0.347
Remnant cholesterol (mmol/L)	0.003 (−0.003; 0.009) *p* = 0.370	0.013 (0.006; 0.019) ***p* < 0.001**	0.046 (0.016; 0.076) ***p* = 0.003**

**Table 3:** Associations between RC and other lipids, one at a time, adjusted for sex, age, BMI, type of surgery, and point of time, all simultaneously. The analyses were performed with a linear mixed regression model and reported as estimated coefficients (B-values) with 95% confidence intervals and *p*-values. Statistically significant *p*-values are given in boldfaced type.

Dependent Variable	Cholesterol B-Value (95% CI) *p*-Value	HDL-c B-Value (95% CI) *p*-Value	LDL-c B-Value (95% CI) *p*-Value	Triglycerides B-Value (95% CI) *p*-Value	ApoB B-Value (95% CI) *p*-Value	ApoA1 B-Value (95% CI) *p*-Value	ApoB/ApoA1 Ratio B-Value (95% CI) *p*-Value
Remnant cholesterol (mmol/L)	0.057 (0.030; 0.083) **<0.001**	−0.232 (−0.306; −0.158) **<0.001**	0.040 (0.010; 0.069) **0.008**	0.279 (0.243; 0.314) **<0.001**	0.220 (0.110; 0.331) **<0.001**	−0.091 (−0.201; 0.020) 0.107	0.289 (0.177; 0.401) **<0.001**

## Data Availability

The raw datasets generated and analyzed during the current study are not publicly available in order to protect participant confidentiality. Case report forms (CRFs) on paper are safely stored. The data were transferred and deidentified to SPSS for statistical analyses. The data files are stored by Innlandet Hospital Trust, Brumunddal, Norway, on a server dedicated to research. The security follows the rules given by the Norwegian Data Protection Authority, P.O. Box 8177 Dep. NO-0034 Oslo, Norway. The data are available on request to the authors.
